# Maternal nutrition literacy and associated factors as determinant of micronutrient supplementation adherence among Chinese pregnant women: a multi-center cross-sectional study

**DOI:** 10.3389/fnut.2026.1757560

**Published:** 2026-03-04

**Authors:** Jia Shi, Ying Wang, Li Pu, Yan Zuo, Jian-jun Zhang, Jing-jing Meng

**Affiliations:** 1 Department of Gynaecology and Obstetrics, The Fourth Affiliated Hospital of Soochow University (Suzhou Dushu Lake Hospital), Suzhou, China; 2 Department of Obstetrics, Zoucheng People's Hospital, Zoucheng, China; 3 Department of Gynecology and Obstetrics Nursing, West China Second University Hospital, Sichuan University, Chengdu, China; 4 West China School of Nursing, Sichuan University, Chengdu, China; 5 Key Laboratory of Birth Defects and Related Diseases of Women and Children (Sichuan University), Ministry of Education, Chengdu, China

**Keywords:** adherence, China, health literacy, maternal health, micronutrient supplementation, nutrition literacy, pregnancy

## Abstract

**Background:**

Maternal nutrition literacy may be a key but underexplored determinant of adherence to recommended micronutrient supplementation during pregnancy in China. This study examined the level of maternal nutrition literacy and its associated factors, and evaluated their relationship with adherence to prescribed micronutrient supplements among pregnant women.

**Methods:**

A multi-center cross-sectional survey was conducted among 471 pregnant women attending antenatal care clinics in three hospitals in Eastern, Southwestern, and Northern China. Maternal nutrition literacy was assessed using the validated Nutrition Literacy Assessment Instrument for Pregnant Women (NLAI-P). Adherence to iron folic acid or multiple micronutrient supplements was measured using 30 day self-reported recall and categorized as adherent (≥80% of prescribed doses) or non-adherent (< 80%). Logistic regression was used to identify independent predictors of adherence.

**Results:**

The mean NLAI-P score was 48.25 ± 10.14 out of 76 (63.5%), with 5.5% of women classified as having excellent nutrition literacy, 58.2% good, and 36.3% poor. Overall, 62.4% of participants were adherent to micronutrient supplementation. Adherence was significantly higher among women with adequate nutrition literacy (good or excellent) compared with those with poor literacy (75.3% vs. 39.8%, *p* < 0.001). In multivariate analysis, adequate nutrition literacy (AOR 3.42, 95% CI 2.15–5.44), older maternal age (AOR 1.09 per year, 95% CI 1.03–1.16), and receiving nutrition information from healthcare providers (AOR 1.85, 95% CI 1.15–2.98) were independent predictors of high adherence.

**Conclusion:**

Maternal nutrition literacy is a strong and modifiable determinant of gestational micronutrient supplementation adherence among Chinese pregnant women. Integrating nutrition literacy assessment and targeted counseling into routine antenatal care may improve supplement adherence and support better maternal and neonatal outcomes.

## Introduction

Maternal nutrition is a cornerstone of public health, directly impacting the wellbeing of both mothers and their offspring. Optimal nutrition during pregnancy ensures proper fetal growth, reduces the risk of adverse birth outcomes, and contributes to the long-term health of both mother and child (1–3). Yet, micronutrient deficiencies remain widespread among pregnant women globally, particularly in low- and middle-income countries (LMICs) (4–6). Iron deficiency, a principal cause of anemia, affects up to 40% of pregnancies worldwide and is notably prevalent in South-East Asia (49%), Africa (46%), and the Eastern Mediterranean (41%) (3, 4, 7). Essential micronutrients such as folate, iron, calcium, vitamin D, and vitamin A are crucial during pregnancy, and deficiencies in these nutrients dramatically increase the risk of complications like maternal mortality, preeclampsia, low birth weight (LBW), preterm birth (PTB), small-for-gestational-age (SGA) infants, stunting, and even infant mortality (6, 8, 9).

In response to this global burden, the WHO and other health bodies recommend routine micronutrient supplementation, such as iron-folic acid (IFA) and, increasingly, multiple micronutrient supplements (MMS), to mitigate these risks (1, 4, 5, 10). Evidence underscores MMS as providing superior benefits to birth outcomes over IFA alone, with notable reductions in LBW, SGA, and neonatal deaths, particularly among undernourished and anemic pregnant women (5, 6). Nevertheless, the positive impacts of supplementation are heavily mediated by the degree of adherence among pregnant women. High adherence to supplementation protocols yields more favorable outcomes: for example, women who adhered to ≥90% of MMS showed a 56 g mean increase in birthweight compared to non-adherent peers, significantly lowering the incidence of adverse birth outcomes (4–6, 10). Despite the proven efficacy, real-world adherence rates to supplementation recommendations remain suboptimal. In some LMICs, only 8% of women who receive or purchase supplements actually consume the recommended dose, severely limiting program impact (11–13). In China, improving adherence has often been approached through general education or antenatal health education, yet these measures do not fully capture whether women can apply nutrition information in daily decisions and sustained supplement-taking (14). Maternal nutrition literacy (NL) represents this applied capacity and remains under examined in the Chinese prenatal context, particularly using pregnancy-specific validated instruments.

This gap in adherence is multifactorial, often rooted in issues of knowledge, accessibility, cultural beliefs, and health literacy (11, 15, 16). Maternal nutrition literacy (NL), the ability to obtain, understand, and apply information about nutrition, is increasingly recognized as a critical determinant of supplementation adherence (12, 13, 17). Nutrition literacy transcends factual knowledge (nutrition knowledge, NK), encompassing broader competencies such as interpreting food labels, critically evaluating sources of information, and enacting behavioral changes (4, 11). However, evidence remains limited in China regarding how pregnancy-specific NL, as a measurable construct distinct from education, relates to real-world micronutrient supplementation adherence during antenatal care. Deficits in maternal nutrition literacy are most pronounced among those with lower educational attainment, reduced financial resources, rural residence, and limited social support (18–20). For instance, higher education was consistently correlated with superior nutrition knowledge and literacy, while multiparity and family influence also positively impacted literacy levels (15, 18, 21). In contrast, limited NL is associated with poorer dietary diversity, later initiation of prenatal care, and increased risk of adverse pregnancy outcomes, including LBW, preterm birth, and stunting (10, 18, 22, 23).

Multiple studies show that nutrition literacy not only enhances dietary practices but also directly promotes behavioral adherence to recommended micronutrient supplementation. Women equipped with higher nutrition literacy are more likely to seek authoritative information, follow medical advice, and recognize the importance of continuous supplementation throughout pregnancy. Interventions aimed at improving maternal nutrition literacy, such as personalized counseling, health education, and community support, demonstrably increase supplementation compliance (10, 18, 24–26). Pregnant women receiving nutrition education are up to 2.8 times more likely to comply with iron-folic acid supplementation and experience greater increases in hemoglobin levels, directly translating to improved pregnancy and birth outcomes (25, 27–29). Moreover, community-based and participatory interventions that combine educational, social, and health system support can attain high compliance rates, frequently exceeding 75–90% in some populations (24, 30, 31).

Socio-demographic determinants, education, socioeconomic status, age, parity, rural vs. urban residence, employment, and the influence of media, collectively predict significant variance in maternal nutrition literacy and, hence, adherence (32–34). Higher family income increases access to health services and reliable information, whereas social support, particularly from partners and family members, enhances understanding and compliance (35, 36). Digital technology and mass media are emerging as important channels, with women possessing greater literacy utilizing internet-based resources more effectively and seeking out professional health advice (37, 38). Conversely, low nutrition literacy commonly results in inconsistent use of supplements, greater susceptibility to misinformation and myths, and diminished capacity to make informed decisions about maternal health (39, 40). Despite the recognized importance of NL, evidence from China is limited on how maternal nutrition literacy shapes adherence to prescribed prenatal micronutrient supplementation in routine antenatal care settings.

This study seeks to examine maternal nutrition literacy and its associated factors as determinants of micronutrient supplementation adherence among pregnant women, aiming to inform future public health interventions and maternal care policies. Our work provides empirical evidence from a large multi-center sample in China, addressing a critical knowledge gap regarding how maternal nutrition literacy shapes real-world adherence to micronutrient supplementation. By identifying both individual and contextual determinants of nutrition literacy and adherence, the findings offer actionable insights for designing targeted education, counseling, and health-system strategies. The results are expected to support policymakers and clinicians in strengthening prenatal nutrition programs and improving maternal and neonatal health outcomes.

## Materials and methods

### Study design and setting

This multi-center, cross-sectional study was conducted between March 9, 2025, and August 10, 2025. To ensure a representative sample with diverse socioeconomic backgrounds, participants were recruited from three distinct medical centers in China: two specialized Obstetrics and Gynecology hospitals located in Suzhou (Eastern China) and Chengdu (Southwestern China), and one comprehensive general hospital in Zoucheng (Northern China).

### Participants and sampling

The study population consisted of pregnant women attending routine antenatal care (ANC) clinics at the selected study sites. A convenience sampling method was employed to recruit participants.

Inclusion Criteria: Pregnant women were eligible for inclusion if they were: (1) aged ≥18 years; (2) at a gestational age of ≥12 weeks; (3) currently prescribed daily oral micronutrient supplements (Iron-Folic Acid or Multiple Micronutrient Supplements); (4) capable of reading and communicating in Chinese; and (5) willing to provide informed consent.

Exclusion Criteria: Women were excluded if they had severe pregnancy complications (e.g., pre-eclampsia requiring hospitalization), pre-existing chronic conditions requiring strict dietary restrictions (e.g., pre-gestational diabetes), or cognitive impairments preventing questionnaire completion.

A total of 553 pregnant women were initially approached and recruited. Following data verification, 82 participants were excluded due to incomplete questionnaires or failure to meet inclusion criteria. The final sample size for analysis was 471 participants.

### Measures

#### Sociodemographic and obstetric characteristics

A structured questionnaire collected data on maternal age, parity, gestational week, marital status, educational level, occupation, household income, and the primary source of nutrition information (e.g., healthcare providers, family, internet).

#### Maternal nutrition literacy (NLAI-P)

Maternal nutrition literacy was assessed using the original Chinese version of the Nutrition Literacy Assessment Instrument for Pregnant Women (NLAI-P), developed and validated by Zhou et al. (20). This instrument has demonstrated satisfactory validity (Scale-level Content Validity Index = 0.98) and reliability (Cronbach's α = 0.82). The NLAI-P consists of 38 items distributed across three dimensions: Knowledge Dimension (23 items): Covers food/nutrition knowledge, balanced diet, weight management, and risk factors for pregnancy complications. Behavior Dimension (7 items): Assesses healthy eating behaviors and lifestyle habits. Skill Dimension (8 items): Evaluates food group analysis, nutrition label reading, and nutrition decision-making.

In scoring, items include multiple-choice and 5-point Likert scales. Each question is scored up to 2 points (2 for correct/positive answer, 0–1 for partial/incorrect answers), resulting in a total possible score of 76 points. Raw scores were converted into percentages. Following the original validation study by Zhou et al. (20), nutrition literacy was categorized using percentage-based thresholds that were defined with reference to China's official health literacy evaluation standard: Excellent (>80% of the total score), Good (60–80%), and Poor (< 60%), rather than ROC-derived thresholds against an external criterion.

#### Micronutrient supplementation adherence

Adherence was measured using a self-reported 30-day recall method. Participants were asked to report the number of days they missed taking their prescribed supplements in the past month. Individual adherence (%) was calculated as: [(30 – number of missed days)/30] × 100. The mean adherence rate was computed as the arithmetic mean of individual adherence percentages across all participants. For categorical analyses, participants with adherence ≥80% (equivalent to taking supplements on ≥24 of the past 30 days) were classified as “Adherent,” whereas those with < 80% were classified as “Non-adherent.”

### Statistical analysis

Data analysis was performed using IBM SPSS Statistics for Windows, Version 26.0 (IBM Corp., Armonk, NY, USA). Continuous variables were tested for normality using the Kolmogorov-Smirnov test and presented as mean ± standard deviation (SD) or median (interquartile range). Categorical variables were expressed as frequencies (*n*) and percentages (%). Bivariate analysis (Chi-square tests for categorical variables and Independent *t*-tests/Mann-Whitney *U*-tests for continuous variables) was used to examine the relationship between NL scores, demographic characteristics, and adherence status. Binary logistic regression analysis was performed to identify independent predictors of supplementation adherence, calculating Adjusted Odds Ratios (AOR) with 95% Confidence Intervals (CI). A *p*-value < 0.05 was considered statistically significant.

We modeled NL in two forms for complementary purposes. For categorical analyses, NL was dichotomized as Adequate (Good/Excellent) vs. Poor to align with the instrument's established cut-offs and to enhance interpretability of the odds ratios. In parallel, NL was also examined as a continuous predictor (per 1-point increase) to assess a dose–response relationship and minimize information loss from categorization. The continuous and categorical NL variables were fitted in separate models. Multicollinearity between NL and education level was assessed using standard diagnostics (variance inflation factor and tolerance), and no problematic multicollinearity was detected.

### Ethical considerations

The study was conducted in accordance with the Declaration of Helsinki. Ethical approval was granted by the Ethics Committee of Dushu Lake Hospital (Approval Number: 250019). Written informed consent was obtained from all participants prior to enrollment.

## Results

### Participant characteristics

A total of 553 pregnant women were approached across the three study sites. After excluding 82 participants (14.8%) due to incomplete questionnaires or failure to meet inclusion criteria, 471 participants were included in the final analysis. The study population was distributed across the three centers: Suzhou (38.2%, *n* = 180), Chengdu (34.0%, *n* = 160), and Zoucheng (27.8%, *n* = 131). The mean age of participants was 29.4 ± 4.3 years. The majority were of Han ethnicity (96.0%) and were married (98.7%). Regarding education, 46.7% (*n* = 220) held a university degree or higher. Approximately half of the participants were nulliparous (52.0%). The demographic and obstetric characteristics of the study population are summarized in Table 1.

**Table 1:** Demographic and obstetric characteristics of the study participants (*N* = 471).

**Characteristics**	**Category**	***n***	**%**
Study site	Suzhou (Eastern China)	180	38.2
	Chengdu (Southwestern China)	160	34.0
	Zoucheng (Northern China)	131	27.8
Age (years)	<25	68	14.4
	25–30	205	43.5
	31–35	148	31.4
	>35	50	10.6
Education level	Junior high school or below	52	11.0
	Senior high school/vocational	199	42.3
	University/college	185	39.3
	Graduate (Master/PhD)	35	7.4
Employment status	Employed	318	67.5
	Unemployed/housewife	153	32.5
Household income (Monthly, RMB)	<5,000	98	20.8
	5,000–10,000	214	45.4
	>10,000	159	33.8
Gestational age	1st trimester (12–13 weeks)	58	12.3
	2nd trimester (14–27 weeks)	205	43.5
	3rd trimester (≥28 weeks)	208	44.2
Parity	Primiparous (0 previous births)	245	52.0
	Multiparous (≥1 previous birth)	226	48.0
Primary nutrition info source	Healthcare provider	268	56.9
	Internet/social media	145	30.8
	Family/friends	58	12.3

### Maternal nutrition literacy (NLAI-P) status

The mean total NLAI-P score for the cohort was 48.25 ± 10.14 (out of a possible 76 points), corresponding to an average percentage score of 63.5%. Based on the standardized cut-offs established by Zhou et al. (20): Excellent NL (>80%): 26 participants (5.5%), Good NL (60–80%): 274 participants (58.2%), and Poor NL (< 60%): 171 participants (36.3%). Analyzing the specific dimensions, participants scored highest in the Knowledge dimension and lowest in the Behavior dimension, indicating a gap between possessing nutritional knowledge and enacting healthy behaviors. The detailed breakdown of scores by dimension is presented in Table 2.

**Table 2:** Scores of NLAI-P and its dimensions among pregnant women (*N* = 471).

**Dimension**	**No. of items**	**Max score**	**Mean score (SD)**	Score rate (%)^*^
Knowledge	23	46	31.28 ± 5.92	68.0%
Behavior	7	14	7.15 ± 2.85	51.1%
Skill	8	16	9.82 ± 3.41	61.4%
Total NLAI-P	38	76	48.25 ± 10.14	63.5%

^*^Score rate calculated as (Mean Score/Max Score) times 100.

### Micronutrient supplementation adherence

Based on the 30-day recall, the mean adherence rate was 81.4% ± 18.6%. The mean adherence rate represents the arithmetic mean of participants' individual adherence percentages, where individual adherence was calculated as [(30 – missed days)/30] × 100. Using the adherence threshold of ≥80% of prescribed doses, 294 participants (62.4%) were classified as Adherent, whereas 177 participants (37.6%) were classified as Non-adherent.

### Factors associated with supplementation adherence (univariate analysis)

Univariate analysis (Chi-square and *t*-tests) revealed significant differences between the Adherent and Non-adherent groups (Table 3). Women in the Adherent group had significantly higher mean NLAI-P scores (51.3 ± 9.2) compared to the Non-adherent group (43.2 ± 9.8, *p* < 0.001). Sociodemographic factors significantly associated with adherence included education level (*p* = 0.002), household income (*p* = 0.015), and the source of nutrition information (*p* = 0.004). Women who relied primarily on healthcare providers for information showed higher adherence rates compared to those relying on the internet or family.

**Table 3:** Univariate analysis of factors associated with micronutrient supplementation adherence.

**Variable**	**Adherent (*n* = 294)**	**Non-adherent (*n* = 177)**	***χ^2^*/*t***	***p*-value**
Age (mean ± SD)	30.1 ± 4.1	28.3 ± 4.4	*t* = 4.38	< 0.001
**Education level**, ***n*** **(%)**			χ2 = 14.82	0.002
Junior high or below	21 (40.4%)	31 (59.6%)		
Senior high	115 (57.8%)	84 (42.2%)		
University	132 (71.4%)	53 (28.6%)		
Graduate	26 (74.3%)	9 (25.7%)		
**Income (monthly RMB)**			χ2 = 8.41	0.015
Low (< 5k)	51 (52.0%)	47 (48.0%)		
Medium (5k−10k)	132 (61.7%)	82 (38.3%)		
High (>10k)	111 (69.8%)	48 (30.2%)		
Total NLAI-P score (mean ± SD)	51.3 ± 9.2	43.2 ± 9.8	*t* = 8.95	< 0.001
**NLAI-P category**, ***n*** **(%)**			χ2 = 41.20	< 0.001
Poor NL (< 60%)	68 (39.8%)	103 (60.2%)		
Good/excellent NL (≥60%)	226 (75.3%)	74 (24.7%)		

**Figure 1:**
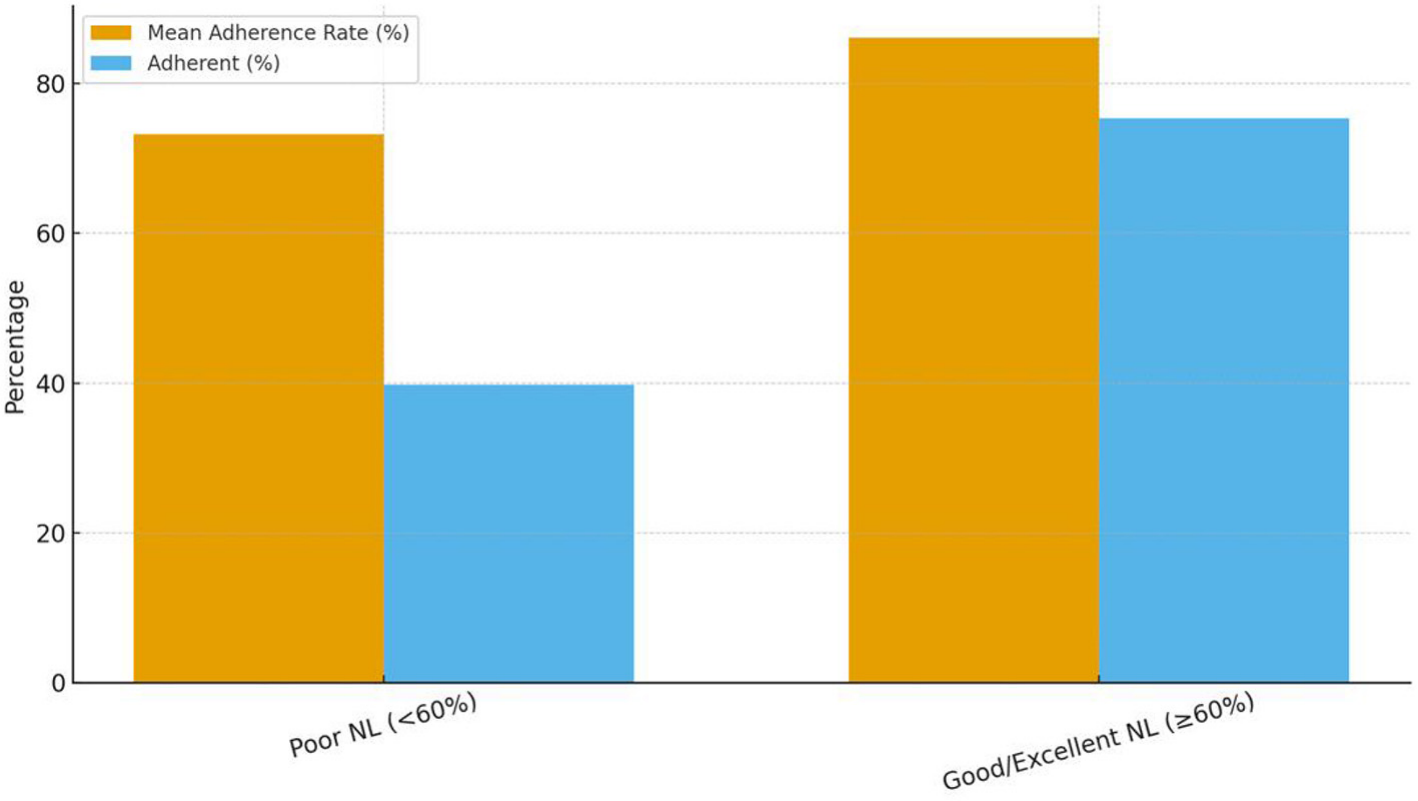
Comparison of mean supplementation adherence rates and adherence status by maternal nutrition literacy level (*N* = 471). This bar chart illustrates the mean self-reported adherence rate to prescribed micronutrient supplements across two categories of maternal NL status, based on the NLAI-P tool. Individual adherence (%) was calculated as [(30 – missed days)/30] × 100, and group mean adherence represents the arithmetic mean of individual adherence percentages within each NL category. Poor NL is defined as an NLAI-P score of < 60% (raw score < 45.6), and Adequate NL is defined as a score ≥60%. The figure shows that women with Adequate NL were significantly more likely to achieve the target adherence threshold of ≥80% (75.3%) compared to women with Poor NL (39.8%), demonstrating a strong behavioral gap mediated by literacy status (*p* < 0.001).

The analysis comparing micronutrient adherence across NL categories revealed a highly significant association between NL status and self-reported supplement intake. As illustrated in Figure 1, women categorized as having Poor NL (score < 60%) demonstrated a significantly lower rate of adherence, with only 39.8% achieving the target of ≥80% prescribed doses (*n* = 68). This starkly contrasts with women categorized as having Adequate NL (score ≥ 60%), where 75.3% were adherent (*n* = 226). The mean adherence rate for the Adequate NL group (86.1%) was substantially higher than the mean rate for the Poor NL group (73.2%). This substantial gap visually confirms that inadequate functional literacy is a major behavioral barrier to maintaining consistent supplementation throughout gestation (*p* < 0.001).

### Determinants of adherence: multivariate logistic regression

A binary logistic regression model was performed to identify independent predictors of supplementation adherence (Table 4). Variables with *p* < 0.20 in the univariate analysis were entered into the model (Age, Education, Income, Parity, Info Source, and NLAI-P Score). After adjusting for confounders, Maternal Nutrition Literacy remained a strong independent predictor of adherence. For every 1-point increase in the NLAI-P total score, the odds of adherence increased by 8% (AOR = 1.08, 95% CI: 1.05–1.11). When categorized, women with Good/Excellent NL were 3.42 times more likely to be adherent compared to those with Poor NL (AOR = 3.42, 95% CI: 2.15–5.44). Other significant predictors included Maternal Age (AOR = 1.09 per year) and relying on Healthcare Providers for information (AOR = 1.85 compared to Internet/Media). Education level was attenuated in the final model, suggesting its effect on adherence is largely mediated through nutrition literacy.

**Table 4:** Multivariate logistic regression analysis of factors associated with high adherence (≥80%).

**Variable**	**β**	**SE**	**Wald χ^2^**	***p*-value**	**AOR (95% CI)**
Age (years)	0.086	0.031	7.69	0.006	1.09 (1.03–1.16)
**Income (ref: low)**			
Medium	0.241	0.285	0.71	0.398	1.27 (0.73–2.22)
High	0.412	0.310	1.76	0.184	1.51 (0.82–2.78)
**Info source (ref: internet)**			
Healthcare provider	0.615	0.244	6.35	0.012	1.85 (1.15–2.98)
Family/friends	−0.154	0.351	0.19	0.661	0.86 (0.43–1.71)
**NLAI-P category (ref: poor)**			
Good/excellent NL	1.229	0.237	26.88	< 0.001	3.42 (2.15–5.44)
Constant	−5.84	1.12	27.1	< 0.001	0.003

Model adjusted for education and parity (not significant in final step).

**Figure 2:**
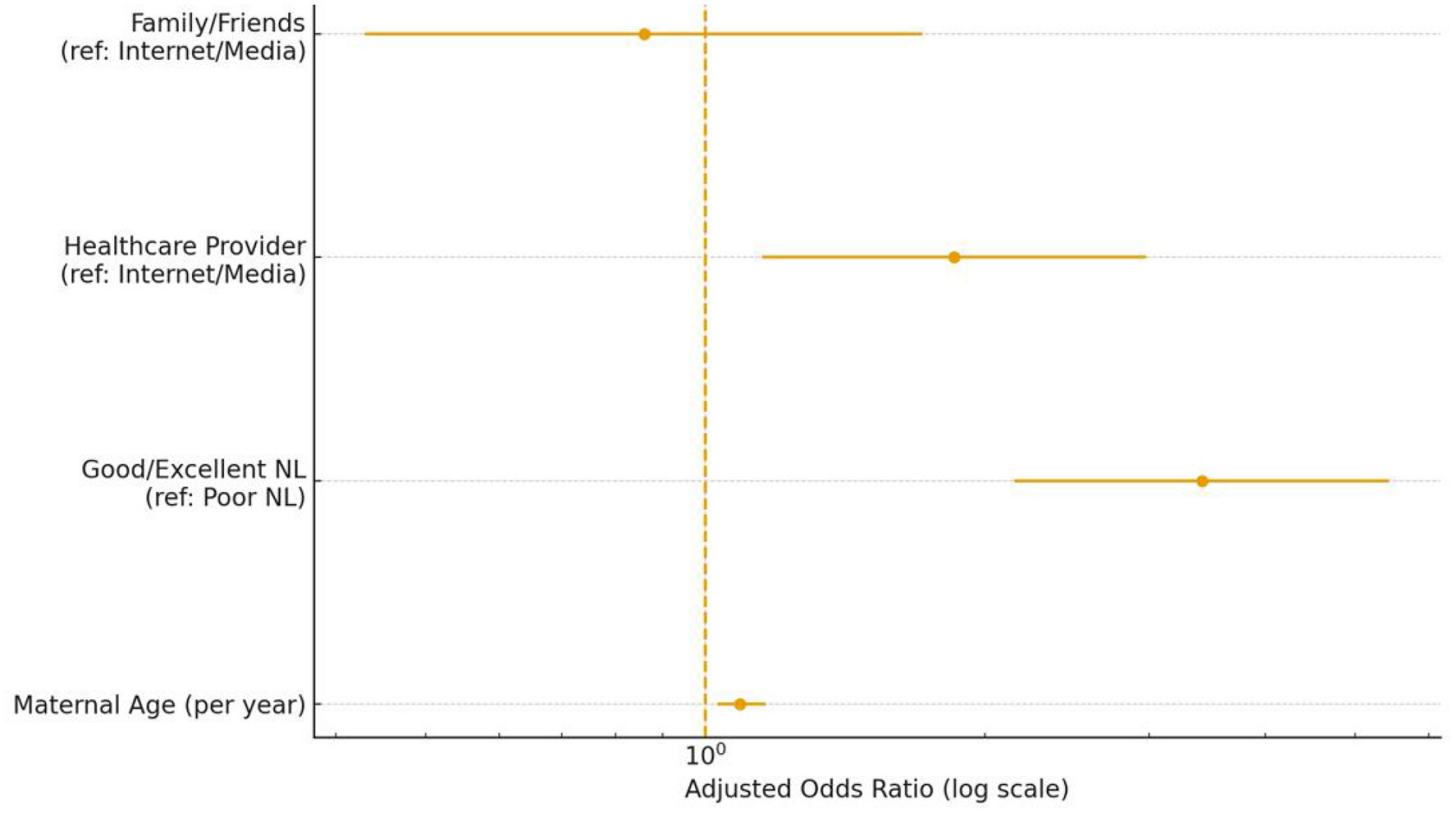
Multivariate logistic regression analysis: independent predictors of high gestational micronutrient supplementation adherence (≥80% Doses). Forest plot displaying the Adjusted Odds Ratios (AOR) and 95% Confidence Intervals (CI) for factors independently associated with achieving high supplementation adherence. The model controls for demographic variables (e.g., income, education). The vertical reference line is set at an Odds Ratio (OR) of 1.0. Variables whose 95% CI does not cross the reference line are statistically significant. Adequate Nutrition Literacy (AOR = 3.42, 95% CI: 2.15–5.44) and reliance on a Healthcare Provider for nutrition information (AOR = 1.85, 95% CI: 1.15–2.98) are the strongest positive predictors of adherence. Maternal Age also showed a significant positive association (AOR = 1.09 per year).

The results of the multivariate binary logistic regression (Figure 2) confirm that Maternal Nutrition Literacy is the strongest independent predictor of high supplementation adherence, even after adjusting for key sociodemographic and behavioral confounders. The model revealed three factors significantly associated with adherence (≥80% doses): NL Level: Women with Adequate NL were 3.42 times more likely to be adherent compared to those with Poor NL (AOR = 3.42; 95% CI: 2.15–5.44). This indicates that functional understanding and skills significantly outweigh other factors in predicting this critical behavior. Maternal Age: Age showed a modest, but significant, positive association, with the odds of adherence increasing by 9% for every one-year increase in age (AOR = 1.09; 95% CI: 1.03–1.16). Information Source: Reliance on Healthcare Providers (doctors/nurses) for nutrition information was highly beneficial, associated with 1.85 times the odds of adherence compared to relying on Internet or social media (AOR = 1.85; 95% CI: 1.15–2.98). Conversely, information received from family or friends was not found to be an independent predictor of adherence in this model (AOR = 0.86; *p* = 0.661).

## Discussion

This multi-center cross-sectional study investigated the status of maternal NL and its association with micronutrient supplementation adherence among pregnant women in China. Our findings reveal that while the majority of participants possessed “Good” nutrition literacy, a significant proportion (36.3%) demonstrated inadequate levels. The overall prevalence of adherence to prescribed micronutrients was 62.4%, indicating a substantial gap in optimal prenatal care. The most significant finding of this study is the robust, independent association between maternal NL and supplementation adherence. After adjusting for confounders, pregnant women with adequate NL were over three times more likely to adhere to their supplementation regimen compared to those with poor NL (AOR = 3.42), underscoring the critical role of health literacy in translating clinical recommendations into daily health behaviors.

Our mean NLAI-P score of 48.25 (63.5%) is slightly higher but comparable to the 46.59 reported by Zhou et al. (20) in the original development and validation of the NLAI-P, suggesting that maternal nutrition literacy (NL) levels in China have remained relatively stable or experienced modest improvement in recent years. Both our study and Zhou et al. (20) found a substantial proportion of pregnant women with inadequate NL, 36.3% in our sample vs. 40.9% in theirs, highlighting a persistent challenge despite ongoing national health initiatives. This enduring prevalence of low NL underscores that a significant demographic of pregnant women remains ill-equipped to process nutrition information, a finding echoed in other studies of Chinese women of reproductive age, which also report low nutrition knowledge and identify education, age, and urban residence as key determinants (41).

While our findings are consistent with Zhou et al. (20) regarding the distribution of NL levels, our slightly higher mean score may reflect incremental improvements, possibly due to increased public health efforts or greater access to nutrition information. However, the gap between knowledge and behavior remains a concern, as highlighted by Zhou et al. (20), who noted that while many women achieve moderate scores in knowledge, far fewer demonstrate adequate nutrition-related behaviors. This gap is further supported by broader literature, which finds that pregnant women often have some awareness of nutrition's importance but lack sufficient knowledge or practical skills to implement dietary recommendations, and that nutrition education from healthcare providers is often perceived as inadequate (29).

Our findings align closely with existing literature highlighting the persistent “knowledge-behavior gap” in maternal nutrition. Similar to our results, which revealed the highest scores in knowledge (68.0%) and the lowest in behavior (51.1%), prior studies have reported comparable discrepancies between awareness and practice. For instance, Rizkia et al. (42) observed that although pregnant women demonstrated adequate nutritional knowledge, behavioral adherence remained low, while Sangwan et al. (43) found that over 90% of women understood the need for additional food during pregnancy but only two-thirds acted on it. This reinforces the widely acknowledged notion that knowledge alone is insufficient to drive meaningful behavioral change during pregnancy. Importantly, this gap can be interpreted in relation to the intermediate performance of the Skill dimension (61.4%), which may represent the practical “translation capacity” required to convert nutrition knowledge into sustained behavior. In the NLAI-P framework, skills include nutrition decision-making and the ability to evaluate and apply information in daily life. In our cohort, common reasons for missed doses included fear of side effects (e.g., nausea/constipation) (28.3%) and perceiving supplements as unnecessary (11.5%), suggesting that the missing link is not awareness but actionable self-management skills (4, 44, 45). We hypothesize that key skills limiting adherence include: (1) managing and troubleshooting mild side effects and timing of intake, (2) interpreting personal need and benefits of supplementation across pregnancy stages, (3) planning routines and using cues or reminders to prevent forgetting, and (4) critically appraising online claims that may undermine confidence in supplementation (15, 45, 46). This may strengthen the rationale for interventions that move beyond knowledge provision toward skills-based, behaviorally anchored counseling.

The literature further attributes this gap to psychosocial and structural barriers that impede the translation of knowledge into practice. Jhaveri et al. (47) emphasized the roles of self-efficacy, family support, and resource availability in influencing dietary behavior, while Kavle (48) and Brink et al. (49) underscored the need for interventions that move beyond didactic education toward personalized, skills-based approaches. Thus, consistent with previous research, our results suggest that effective maternal nutrition interventions must target behavioral competence and environmental support systems to close the gap between “knowing” and “doing.”

The adherence rate of 62.4% reported in our study is consistent with recent evidence indicating moderate but suboptimal compliance with iron and folic acid supplementation among pregnant women. For instance, Tegodan et al. (50) found a nearly identical adherence rate of 62.3% among Ethiopian women, emphasizing that factors such as health literacy, exposure to nutritional counseling, and forgetfulness are critical determinants of supplement use. Similarly, studies in other regions have reported lower adherence levels, ranging from 40.9% in Adwa town Gebremichael and Welesamuel (51) to 51.4% among pastoralist women Boti et al. (52), demonstrating that adherence remains a global challenge. In China, available evidence suggests that guideline-concordant supplement use is often suboptimal, although operational definitions differ across studies. In a Beijing cohort, 97.2% of women reported taking folic acid supplements, yet only 24.2% were compliant with the recommended dose and duration, and even under a less stringent criterion the correct-use rate was 40.3%. In rural Shaanxi, only 44.5% of women reported periconceptional folic acid use despite relatively high pick-up rates (53). Taken together, our observed adherence rate of 62.4% (defined as taking ≥80% of prescribed doses over the past 30 days) may reflect a moderate level of adherence in an urban/suburban ANC population. However, direct comparisons across Chinese studies should be interpreted cautiously because of differences in supplements assessed and adherence definitions (30-day dose-taking vs. periconceptional “correct use”).

In contrast to earlier work that primarily emphasized formal education as a determinant of adherence Agegnehu et al. (54), our analysis identified *nutrition literacy (NL)* as a more potent predictor. This finding aligns with the emerging conceptual distinction between general education and applied health literacy, where NL reflects the ability to access, evaluate, and utilize nutritional information effectively. Studies such as Anato and Reshid (55) and Ramachandran et al. (56) support this notion, showing that targeted nutrition education significantly improves adherence and hemoglobin outcomes through enhanced comprehension and self-efficacy. Our results further demonstrate that women with higher NL may be better equipped to manage minor side effects, such as nausea, which accounted for 28.3% of non-adherence in our cohort, reflecting critical literacy and self-regulatory capacity. Together, these findings highlight the necessity of integrating nutrition literacy development into antenatal education to close the persistent gap between knowledge acquisition and behavioral adherence.

Our findings that women who relied primarily on healthcare providers (56.9%) exhibited higher adherence to supplementation than those depending on online sources are consistent with current literature emphasizing the enduring value of professional guidance in maternal nutrition. Multiple studies confirm that healthcare providers remain the most trusted and influential source of dietary advice during pregnancy. For instance, Gjestvang and Haakstad (57) found that although the Internet is widely used (up to 97%), healthcare professionals (HCPs) were consistently rated as the most reliable source of information and were associated with better adherence to nutritional guidelines. Similarly, Mitran et al. (34) demonstrated that advice from healthcare providers significantly improved diet quality and supplement adherence among women of reproductive age, underscoring the mediating role of professional counseling in shaping nutrition behaviors.

Conversely, reliance on digital and social media sources has been linked to confusion and misinformation. Recent evaluations of online pregnancy nutrition portals found considerable inconsistencies in supplement recommendations and poor alignment with official guidelines Skowrońska et al. (58). Similarly, qualitative research has shown that while pregnant women often turn to the internet due to convenience or lack of provider engagement, this self-directed information seeking can lead to anxiety and conflicting interpretations Snyder et al. (59). These findings support Zhou et al.'s (20) conceptualization of the “judgment of nutrition information” as a core skill within the Nutrition Literacy Assessment Instrument for Pregnant Women (NLAI-P), highlighting the importance of critical evaluation of information sources.

### Strengths, limitations, and future directions

This study has several strengths. First, a key strength of this study is the relatively large sample size and multi-center recruitment across three urban/suburban hospitals, which increases statistical precision and reduces the likelihood that findings are driven by site-specific practices compared with many single-site studies in this field. Second, it utilized the NLAI-P, a tool specifically validated for Chinese pregnant women with high content validity (0.98) and reliability (Cronbach's α = 0.82), ensuring the measurement was culturally and contextually appropriate. Third, the multi-center design, covering Eastern, Southwestern, and Northern China, improves the generalizability of the findings compared to single-center studies. However, limitations must be acknowledged. First, the cross-sectional design precludes causal inference; while we observed a strong association, we cannot definitively state that improving NL causes increased adherence. Second, adherence measured using the self-reported 30-day recall method is susceptible to recall error and social desirability bias. As a result, adherence may have been overestimated in this study. If participants with higher nutrition literacy were also more likely to report socially desirable behaviors, the strength of the observed association between nutrition literacy and adherence may have been inflated. Future studies should consider incorporating more objective adherence measures, such as pill counts, blister pack returns, pharmacy refill records, or electronic monitoring, to reduce reporting bias and strengthen causal inference. Third, convenience sampling from ANC clinics in three urban and suburban hospitals may limit the generalizability of the findings. Our sample was socio-demographically advantaged, with a high proportion of university-educated (46.7%), married (98.7%), and Han ethnicity (96.0%) women, which may overestimate both nutrition literacy and micronutrient supplementation adherence relative to the broader pregnant population. Accordingly, the associations observed in this study should not be generalized to key at-risk populations, including rural pregnant women, women with lower educational attainment, ethnic minority groups, migrant pregnant women, and those facing socioeconomic disadvantage, who may have lower nutrition literacy and greater structural barriers to adherence (e.g., limited access to antenatal education, reduced continuity of care, and financial or time constraints). Future studies should use probability-based or multi-stage sampling across diverse settings and intentionally recruit underrepresented groups to test whether the magnitude of the nutrition literacy–adherence association differs across social and geographic strata.

The strong link between NL and adherence suggests that standard antenatal care must evolve beyond simple prescription. Clinicians should screen for low nutrition literacy using brief tools derived from the NLAI-P. For women identified as having low NL, simple “take this pill” instructions are insufficient. Interventions should focus on the Skill and Behavior dimensions, because they directly address the observed knowledge–behavior gap. Practical components should include side-effect management strategies, individualized risk–benefit communication to address “unnecessary” perceptions, and simple adherence planning tools (reminder systems and routines), alongside guidance for evaluating online nutrition information. Furthermore, since education level is a fixed demographic, targeting modifiable NL through hospital-based education programs offers a practical pathway to improving maternal and child health outcomes. Additionally, future studies should prioritize rural and underserved populations and apply sampling strategies that better capture variability in nutrition literacy and adherence barriers.

## Conclusion

Nutrition knowledge among Chinese pregnant women is relatively higher than behavior, indicating that knowing recommended practices does not reliably translate into consistent supplement-taking. Improving pregnancy outcomes may therefore require interventions that move beyond information provision to strengthen functional and critical nutrition literacy, including practical skills for applying guidance and evaluating the reliability of digital nutrition information. Our findings can inform the design of targeted antenatal education programs and health system strategies to improve micronutrient supplementation adherence, particularly among women who are socioeconomically disadvantaged or who rely heavily on unverified online sources.

## Data Availability

The raw data supporting the conclusions of this article will be made available by the authors, without undue reservation.
